# The zero-emissions resource pool: construction materials compatible with a realistic view of delivering zero-emissions in the UK by 2050

**DOI:** 10.1007/s44150-025-00171-1

**Published:** 2025-09-04

**Authors:** Charlotte Taylor, Julian M. Allwood, Takuma Watari, Will Hawkins

**Affiliations:** 1 Department of Architecture and Civil Engineering, University of Bath, Bath, UK; 2 Department of Engineering, University of Cambridge, Cambridge, UK; 3 Materials Cycles Division, National Institute for Environmental Studies, Tsukuba, Japan

**Keywords:** Decarbonisation, Construction materials, Zero-emissions infrastructure, Embodied carbon

## Abstract

The construction sector faces the daunting task of meeting growing construction demand with a 'zero-emission resource pool'—materials that are compatible with a near-future zero-emissions economy. Most decarbonisation roadmaps and scenario analyses for the sector depend heavily on high-risk technologies such as carbon storage that have not yet been deployed at significant scale, or favour recycling whilst overlooking likely constraints from limited supplies of zero-emissions electricity. This paper therefore provides a first critical review of options to supply construction materials in the UK with realistic expectations about the availability of carbon storage, zero-emissions electricity and zero-emissions transport. The paper focuses on nine key construction materials—concrete, steel, aluminium, structural glass, timber, earth, stone, lime and straw. We conclude that the zero-emissions resource pool includes virgin bio-based materials, limited by the availability of productive land, virgin earth and stone, limited by local geology and transportation, recycled materials, limited by the availability of scrap and emission-free electricity, and reused components, limited by availability and refurbishment potential. This points to the need for a revision to the national construction strategy and a range of entrepreneurial opportunities in delivering the services of construction within a reduced material budget.

## Introduction

Globally agreed climate targets aim at halving global greenhouse gas (GHG) emissions by 2030 and reaching zero by 2050 [1]. In response, a growing number of countries, including the UK, have pledged to reduce their national emissions to zero by mid-century [2]. Whilst notable progress is being made in several sectors, such as electricity and transport, the construction sector presents one of the most daunting challenges in meeting this target. Currently, the construction sector in the UK accounts for 25% of national GHG emissions, a quarter of which arises in material production [3] used in key building elements such as foundations, frames and other forms of superstructure [4]. Delivering on the UK’s legally binding emissions-target will therefore require alternative construction materials and reduced demand.

**Fig. 1:**
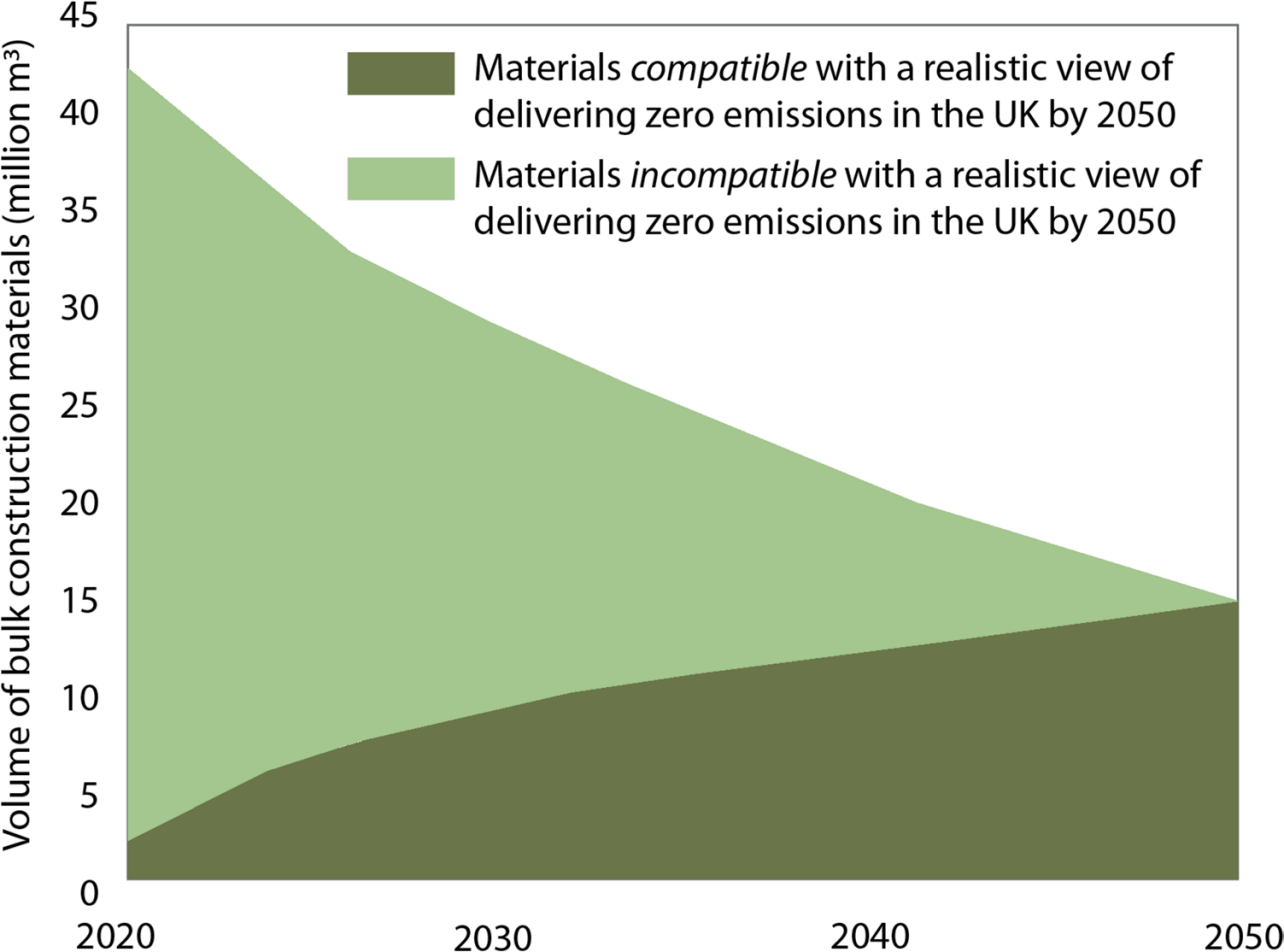
Graphical description of how demand reduction and an increase in the supply of materials compatible with a realistic view of delivering zero-emissions in the UK can lead to decarbonisation of the construction materials sector (Adapted from UK FIRES ‘Construction Sector Innovation within Absolute Zero’ report) [9]

Previous studies have investigated the roles of material efficiency improvements [5, 6] and fuel-switching [7, 8] to support decarbonisation of construction material supplies. Whilst essential, transformative, and creating rich innovation potential, efficiency improvements will not be sufficient to meet global and UK emissions targets [9]. Options for switching energy-intensive processes away from current fossil fuel energy sources include hydrogen and carbon capture, utilisation and storage (CCUS) technologies which are relied upon in industry roadmaps but are not currently deployed economically or at scale [10]. Furthermore, although hydrogen might replace fossil fuels as an energy source for many hard-to-decarbonise materials [11–13], or residual emissions could be reduced through CCUS, uncertain deployment rates for these technologies pose a critical risk to delivering on the 2050 target. To reduce the risks of achieving the target, the use of materials incompatible with zero-emissions production must be reduced and the zero-emissions material pool expanded, as illustrated in Fig. 1, and it is likely that this requires a reduction in demand [9]. The importance of material substitution and the transition to a zero-emissions circular economy has been highlighted in several studies [14, 15]. However, while these analyses provide important information for formulating national material production strategies, key risks including dependence on national capacity for zero-emissions energy supply have been ignored [16].

The scale and mix of zero-emissions construction materials likely to be available in the UK by 2050 is as yet unknown. However, before industrialisation and the widespread use of fossil fuels, material supplies were largely delivered from sources such as quarries, forests and fields [17]. Early material production processes were relatively uncomplicated, for example, harvesting stone and timber, or mixing and heating bricks. The materials of these early processes are now produced more efficiently than in pre-industrial days, with energy intensities of the order of 1–5 MJ/kg [18]. However, other industrial construction materials such as aluminium and steel have energy intensities up to ~ 210 MJ/kg [19]. Generally, material production is more energy intensive if it requires smelting or heating to high temperatures [20]. As a result, several recent studies have explored the use of construction materials with lower embodied energy such as timber [21–23], whilst others have analysed supplies from recycling and reuse [24, 25].

**Fig. 2:**
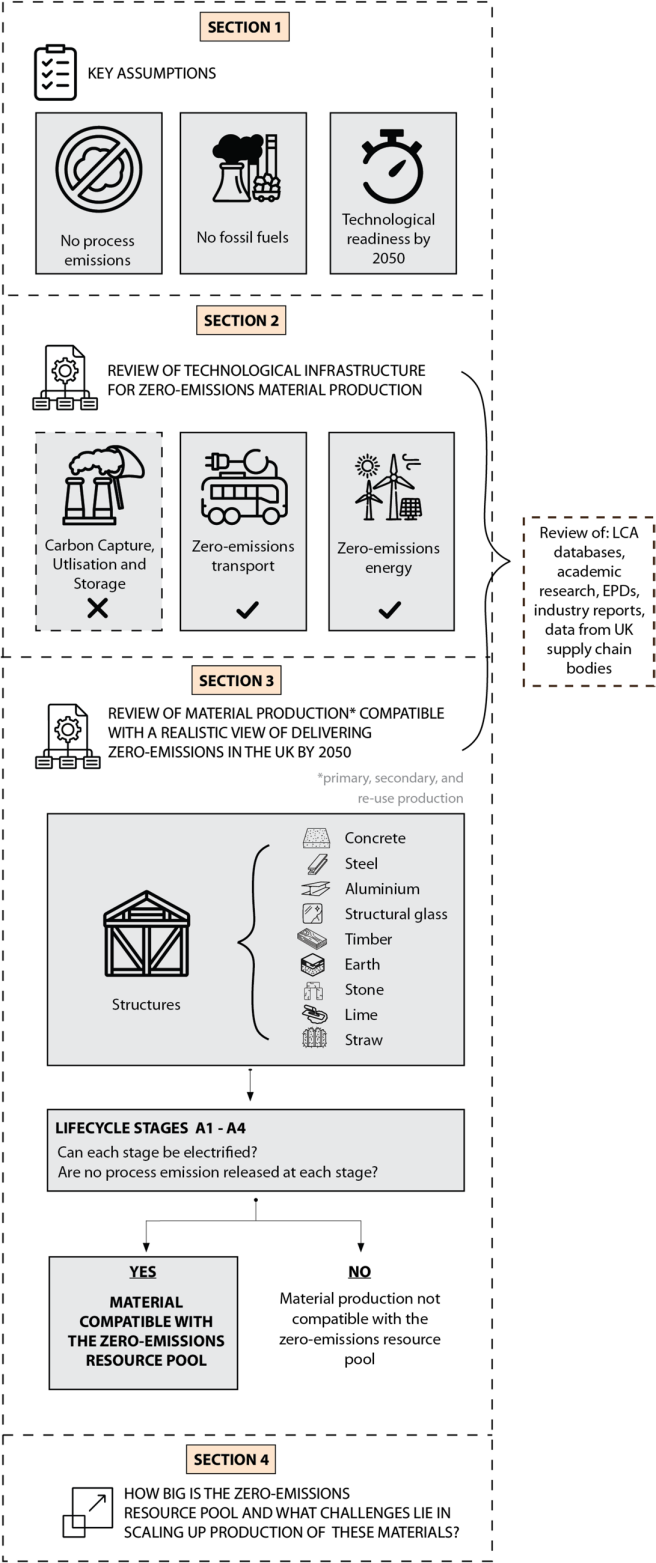
Overview diagram of the review showing contents and approach of each section

This paper aims to provide a comprehensive review of near-future (until 2050) options for the zero-emissions supply of construction materials in the UK including a full array of bio-based and circular materials. The review aims to span the full range of decarbonisation strategies, accounting for uncertainties in build rates of new processes. An overview of the review is described in Fig. 2 and below.

Section"Technological infrastructure for zero-emissions material production"considers three key resources relied upon in industry roadmaps [12, 13, 26, 27] to support zero-emissions material production: CCUS, zero-emissions energy and zero-emissions transport. Novel processes for delivering these resources are evaluated against the requirement that they scale significantly by 2050. In Section"Material production compatible with a realistic view of delivering zero-emissions in the UK by 2050", we focus on the production of construction materials, recognising that the supply of the resources discussed in Section"Technological infrastructure for zero-emissions material production"may constrain production. The section provides a detailed review of options for primary and secondary production of nine construction materials that are either used at scale in the UK today or were important in the past [17]: concrete, steel, aluminium, structural glass, timber, earth, stone, lime and straw. These materials were selected based on their contribution to embodied carbon in UK construction—concrete, steel, timber, clay products, natural stone, gypsum products, glass, and aluminium collectively account for the highest material-related emissions by mass [28]. Straw is included as a vernacular material with demonstrated structural applications for modern typologies [29]. If the material can be produced without emissions and there is sufficient technological infrastructure to anticipate meaningful scale of production by 2050, then the material is considered compatible with zero-emissions goals. Section"Discussion"discusses the consequences of this realistic articulation of a zero-emissions resource pool for construction, in particular exploring supply options that have been under-prioritised and options to deliver construction with a constrained material supply.

### Zero-emissions terminology

In this paper, we use the term *zero-emissions* to refer to the production of construction materials that involves no fossil fuel use and does not result in any greenhouse gas (GHG) emissions during processing. We intentionally avoid the term *net-zero*, as it often introduces ambiguity—specifically, whether the material was produced using fossil fuel–intensive methods and subsequently offset, or whether it was genuinely produced without emissions.

## Technological infrastructure for zero-emissions material production

The embodied emissions from existing primary materials production processes primarily arise from fuel combustion to reach high temperatures, from emissions released as part of chemical reactions and from the transport used to move materials from sites of extraction to the site of manufacture. The first of these could potentially be decarbonised with electrical heat, although some higher temperatures remain difficult. However, even with electrical heat, process emissions (where present) would not be avoided. Decarbonisation of these emissions therefore depends either on carbon capture and storage, or on using different production processes, for example using hydrogen as a reductant for iron ore. However, without a native supply of hydrogen, it must be produced by electrolysis or with carbon capture. As a result, the potential scale of alternative primary production is constrained by the future supply of *zero-emissions energy and carbon storage*. Due to the high volumes required for major projects, a further constraint for construction materials is the provision of *zero-emissions materials transport*. This section reviews the likely scalability of these three resources, to set the scene for analysis of the options to decarbonise the nine major material groups.

### Carbon capture, utilisation and storage

The phrase “Carbon Capture Utilisation and Storage (CCUS)” refers to a suite of potential technologies that might capture CO_2_ emissions from large point sources such as power plants, refineries or blast furnaces, or even be linked to direct capture of atmospheric CO_2_ [30]. This ‘breakthrough’ technology has been in development since the 1970 s [31], but still faces technical, economic, and social uncertainty. After 50 years of development, global capacity for these technologies has reached just 0.08% of global emissions and is growing at just 0.004%/year [32]. Despite this, CCUS continues to be described as a key element of the UK’s net-zero strategy, which anticipates 20–30 MtCO_2_ captured and stored by 2030 [33] with little discussion of the risk of deployment shortfall.

**Fig. 3:**
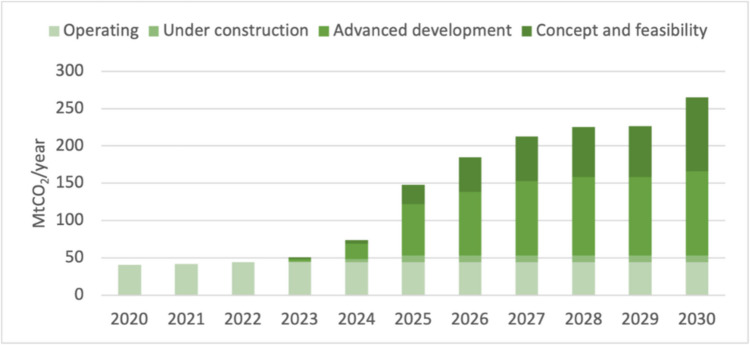
Capacity of global large-scale CO_2_ capture projects, current and planned vs. the Net Zero Scenario, 2020–2030, adapted from IEA [36] (n.b. there is currently no CCS capacity in the UK)

Figure 3 shows that operating CCUS capacity at the global scale has increased little in recent years with only a small increase ‘under construction’ [34]. However, this reality is often ignored in climate policy analyses. For example, the 2010 IEA report assumed that CO_2_ capture for just the steel and cement sectors would reach around 195 Mt-CO_2_ in 2021 but the current global operating capacity for the steel and cement sectors is just under 1 Mt-CO_2_ [35]. Note here that these figures only account for CO_2_ *capture*, without *storage*. Whilst the latter would refer to something relatively permanent (i.e. stored in deep geological formations), if CO_2_ is only captured, then it is likely to be stored in containers temporarily before being used for some kind of industrial application which may or may not end up in the re-release of the CO_2_ into the atmosphere [35].

As a result, the over-reliance on uncertain CCUS technologies in the UK’s strategy increases the likelihood that net zero targets will be missed. Therefore, any CCUS capacity deployed in the UK by 2050 is likely to be far short of anticipated demand. Consequently, this study assumes that:

- There will be no supply of CCUS at scale available for construction material production.
- Any process with continuing fossil fuel or process emissions is incompatible with a realistic view of delivering zero-emissions in the UK by 2050.

### Zero-emissions energy

Fossil fuels currently supply 80% of energy demand in the UK, directly or indirectly [37]. The national energy mix in 2023 was largely dominated by primary oil (crude oil and Natural Gas Liquids) (38% of total production), followed by natural gas (33%), electricity (consisting of nuclear, wind, solar and hydro) (17%), bioenergy and waste (12%), and coal (0.4%) [38]. Without CCUS, there is no option to continue generating energy from fossil fuels. Instead, electricity must be supplied from zero-emissions sources and any use of fossil fuels for heating and transport must be replaced. Despite ambitious plans to deliver up to 50GW of wind power by 2030, becoming “the Saudi Arabia of wind power”, and 70GW solar power by 2035, the UK currently has only 12.7GW of wind capacity and 14GW of solar capacity [10]. The mega-projects of expanding supply require long and complex, pre-construction societal discussions about public funding, land rights [39], impacts on local communities [40], safety [41], public perception [42], legal and environmental compliance and the complicated processes of commercial contracting [43]. As a result, there will likely be a shortfall in zero-emissions energy supply in the UK by 2050 [44], and at current growth rates, such supply will likely meet only 60% of anticipated electricity demand [45].

Bioenergy could, potentially, complement zero-emissions electricity but it is difficult to expand the supply sustainably [46, 47]. Most electricity from bioenergy (almost 9% of electricity generated in the UK in 2021) comes from plant-based biomass, often in the form of wood pellets and although the UK produces around 66% of its biomass supply, it is the largest wood pellet importer in the world. Modelling carried out for the 2023 Net Zero Growth Plan [48] suggests that bioenergy demand could be in the range of 25-35TWh (between 8% and 11.4% of the energy generated in the UK in 2021) between 2035–2050 and the Biomass Strategy 2023 [49] states that these demands could be met by 2050. However, the potential expansion is calculated from technical potentials and does not account for the time needed to scale up businesses and supply chains or competing uses of land. For example, with anticipated increases in food demand [50] any increase in bioenergy supply may compete with food security [51]. It is therefore unlikely that there will be a substantial global increase in bioenergy supply by 2050, even if the suggested technical potentials are achieved, and this limits the likelihood of meeting bioenergy targets.

An emerging body of literature focuses on hydrogen technologies, including the production of direct reduced iron (DRI) using green hydrogen [52, 53]. Zero-emissions ‘blue’ hydrogen can be made using conventional steam reforming linked to CCUS, but Section"Carbon Capture, Utilisation and Storage"has discussed the high risk of assuming any significant capacity. Production of ‘green’ hydrogen requires a large supply of zero-emissions electricity, a predicted shortfall of which will limit its production. Several studies have identified the risks of relying on rapid technological deployment of green hydrogen [54–56]. For instance, Odenweller et al. [57] demonstrate that despite strong momentum and enthusiasm around green hydrogen, the market ramp-up of electrolysis is a decisive bottleneck on the route to achieving climate targets and there is a risk of a long-term gap between likely supply and demand. The challenge here is two-fold: on the one hand infrastructure deployment inevitably takes a long time, due to the realities of engineering and construction, including the political and regulatory procedure; on the other hand, electrolysis requires a very large surplus of zero-emissions electricity [58]. The UK Government’s Net Zero Strategy[59] aims to install 5GW of hydrogen capacity by 2030, which would be enough to deliver 42TWh energy, yet this is not under construction and is less than a quarter of the current demand from hard-to-decarbonise sectors including aviation, shipping and high-grade industrial heat [45].

These findings draw attention to the reality that zero-emissions electrical supply is unlikely to grow fast enough to match growing demand on the path to zero-emissions. Therefore, material production in 2050 will be limited by a constrained supply of zero-emissions electricity.

### Zero-emissions transport

Growing demand for transport, from local to transnational scales, increases emissions, air pollution and energy demand [60]. Today, 81% of domestic freight is moved by road, 12% by water and 7% by rail [61]. Although the transition to electric cars in the UK is well underway [62], the decarbonisation of Heavy Goods Vehicles (HGVs) is still insignificant. These vehicles are used extensively to move construction materials, and although prototype vehicles have been developed, they require significant electrical power and storage [63]. Despite this, the UK government has pledged to end the sale of non-zero-emissions HGVs by 2040, with those 26 T or less being phased out by 2035 [64]. Even if a fully electric fleet is on the roads by this date, a recent study [65] has forecast that by 2050 the sector will only have 60% of the electricity required to power a fleet equivalent to that in use today, suggesting that an equivalent cut in mileage is required unless substantial energy improvements are made.

Currently, there are no zero-emissions freight ships in UK waters, so there is an urgent need for exploration of means to electrify ship power [66]; without zero-emissions shipping, an enormous expansion in international rail capacity could be required. Still, only 38% of the UK’s rail network is electrified and estimates show that current rates of railway track electrification would need to be increased eightfold to meet the net zero target by 2050 [67]*.* Zero-emissions aviation is at an early stage of development, with battery-powered flight only feasible for short journeys with small aircraft. Hydrogen is considered one possibility for longer flights but remains unused at scale and subject to the fuel supply constraints previously discussed. Biofuels for aviation are closer to technological readiness but are similarly constrained by resource availability and competing demands. As a result, the industry could face a rapid contraction [68].

Therefore, there is limited availability of international zero-emissions transportation and in an zero-emissions economy, modes of mass transport may be limited to the territorial boundary of the UK or international goods could become a high-value, scarce resource, increasing incentives to meet needs and use resources locally.

## Material production compatible with a realistic view of delivering zero-emissions in the UK by 2050

**Fig. 4:**
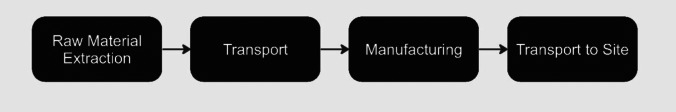
Schematic of the scope of life cycle stages covered in this section

This section reviews the options for zero-emissions production of key construction materials in the UK. Historic and current demand for each material is first presented, along with a schematic description of possible production processes. Decarbonisation strategies for each material are then reviewed to determine to what extent the material is compatible with a realistic view of delivering zero-emissions in the UK by 2050. Building on the analysis of Section"Technological infrastructure for zero-emissions material production", this evaluation spans the four life-cycle steps illustrated in Fig. 4, determining whether each stage can be electrified or completed without process emissions, at the UK scale by 2050. The vulnerability of each primary production route to uncertain CCUS and hydrogen deployment is also assessed, along with the production routes of recycling and reuse options. Since materials requiring fossil carbon are deemed incompatible with a realistic pathway to UK zero-emissions by 2050, relative CO_2_ reductions are not the focus. Instead, energy intensity is reviewed as a more relevant metric in a future of constrained renewable energy, with energy intensities summarised in Section "Conclusion", Table 1. The details of practical construction methods of each material are out side of the scope of this review.

### Concrete

**Fig. 5:**
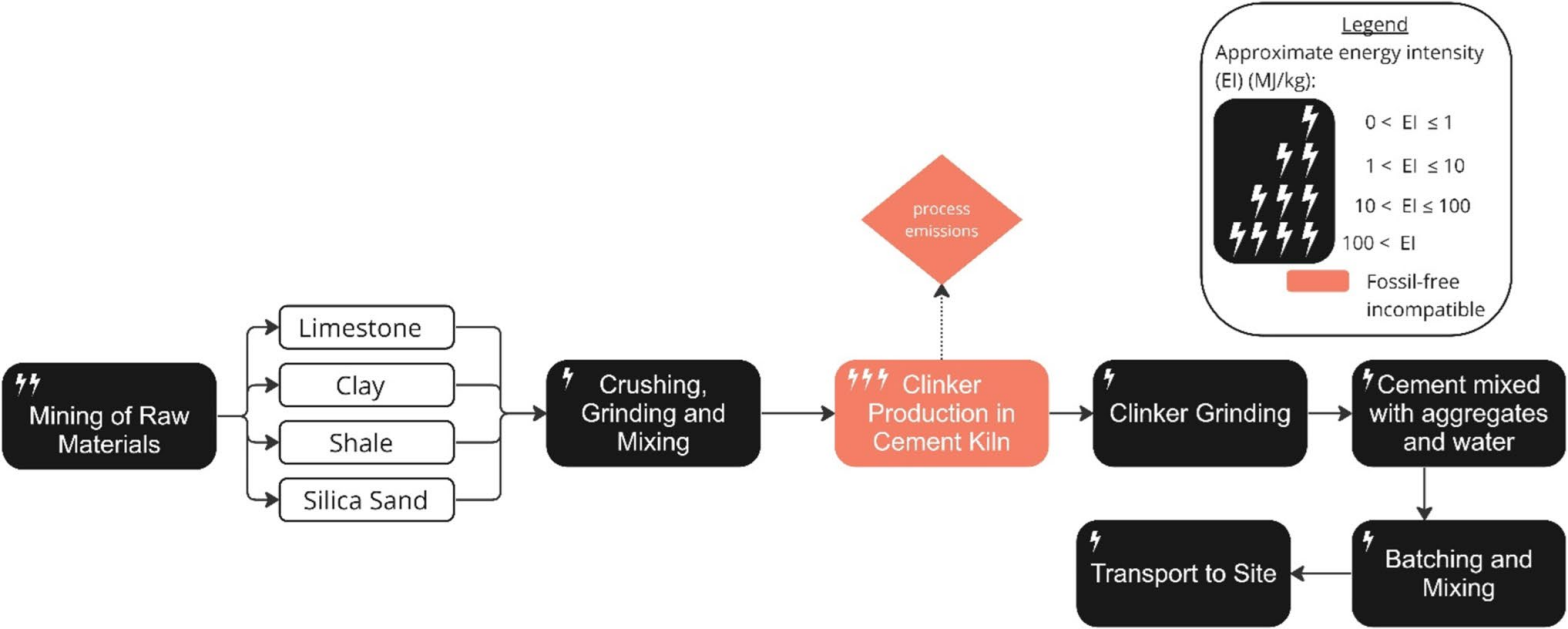
Process map of primary concrete production, adapted from (Ruijven et al. 2016) and (Martinet et al. 2023). The approximate energy intensity of each process in the production chain, and the process’ compatibility with a realistic view of delivering zero-emissions by 2050, is indicated (see figure legend) [72, 73]

Concrete is the dominant material of modern construction, used extensively in infrastructure and across all building forms. In 2020, the UK cement industry was responsible for 7.3 million tons of CO_2_, approximately 1.5 per cent of total domestic CO_2_ emissions [69] and 9 per cent of the UK’s manufacturing emissions [70]. Out of the seven stages in the manufacture of Portland cement described in Fig. 5, around 50% of cement production emissions come from calcination in clinker production, around 40% from fuel combustion to provide heat (up to 1,450 °C), and the rest from electricity use and transport [69]. While the mineral processing operations of cement production are energy-intensive, the unavoidable CO_2_ emissions of carbonate calcination present the greatest challenge to the goal of decarbonising construction materials [71]. If cement is to be part of a future zero-emissions resource pool, existing Portland cement production must be replaced by different, scalable, economically viable processes.

A substantial body of research has studied the options for decarbonising the production of cement, achieving ‘net zero’ through a combination of supply-side measures. These include thermal and electric efficiency [74], kiln electrification [75], use of alternative binders [76] and use of Supplementary Cementitious Materials (SCMs) [77]. One promising alternative binder to Portland Cement is Alkali-activated cements (AACs), which use alkaline solutions like sodium hydroxide or sodium silicate to activate ground granulated blast furnace slag (GGBS) and pulverised fly ash (PFA) [78]. However, because sodium silicate is made by fusing silica sand with sodium carbonate at over 1,000 °C, and sodium hydroxide through the energy-intensive chlor-alkali process [79], their production is heavily reliant on fossil fuels, limiting the potential for fossil-free production without significant advancements in electrified high-temperature processes or alternative low-carbon methods. The most common SCMs used today are ground granulated blast furnace slag (GGBS) and pulverised fly ash (PFA) which are by-products of blast furnaces or coal-fired power stations, respectively, both of which can only be decarbonised through CCUS, making them incompatible with a realistic view of delivering zero-emissions in the UK by 2050 [9].

One key option is calcined clay, produced by calcination of kaolinite. Although energy-intensive, the process can be electrified [80] and avoids process emissions [73]. However, to maintain sufficient early strength, workability, and durability, calcined clay can be used only to substitute a maximum of 50% of Portland clinker [73].

A recent pioneering technology—Cambridge Electric Cement (e-cement)—uses recycled cement paste as a precursor and is the first zero-emissions cement in the world to be trialled at a pilot scale [81]. However, while promising in terms of decarbonisation, it is as yet unknown how rapidly it can scale. Therefore, e-cement is not compatible with the framework which assumes zero-emissions by 2050.

As a result, in a zero-emissions economy, large-scale low-cost use of concrete is unlikely to be feasible before 2050. Today, most literature listing the available pathways for cleaner construction do not include the reuse of concrete pieces in new buildings as a strategy to consider. However, Kupfer C. et al. (2023) performed a critical review of 77 precedents of concrete re-use in new construction projects, highlighting the potential for greater circularity, albeit with certain challenges [82]. Their findings emphasise the need for improved knowledge of the existing building stock and historical construction methods, an expanded set of proven design solutions and connection details, and greater integration across disciplinary boundaries. Due to its ubiquity and diverse uses, finding zero-emissions alternatives to concrete is likely to be a dominant challenge in decarbonising construction.

### Steel

**Fig. 6:**
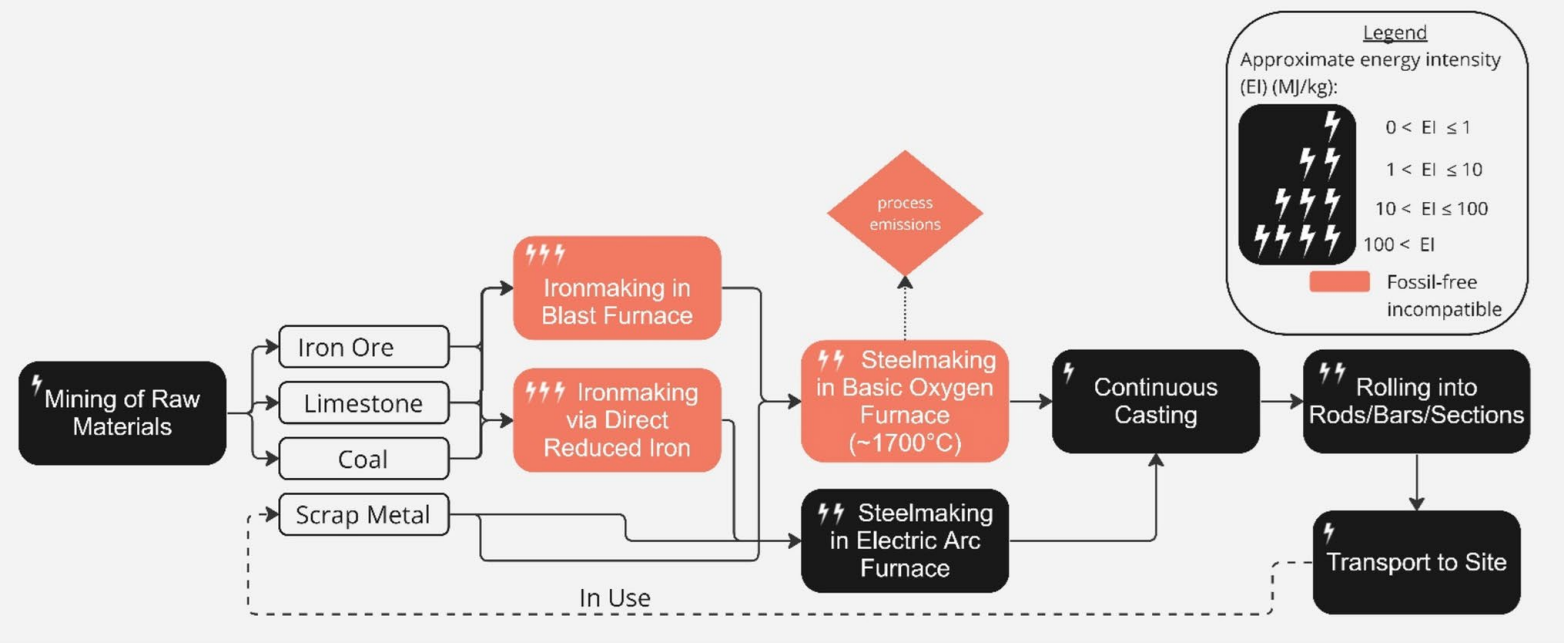
Process map of primary and secondary (via ‘Scrap Metal’ route) steel production, adapted from (Pimm et al. 2021), (Devlin et al. 2022) and (Griffin et al. 2019). The approximate energy intensity of each process in the production chain, and the process’ compatibility with a realistic view of delivering zero-emissions by 2050, is indicated (see figure legend) [52, 83, 88]

Steelmaking is the largest industrial sector in the UK measured by energy demand or GHG emissions, accounting for 26% of British industrial emissions [83]. Currently, the dominant steel manufacturing process for primary steel is via the ore-based blast furnace/basic oxygen furnace route (BF/BOF), as described in Fig. 6. Primary steel production via the BF/BOF could be decarbonised only by CCUS. The International Energy Agency (IEA) expects that, on average, 40% of 2050 emissions reduction for the steel sector will come from CCUS. This trend is reflected in the academic literature on steel sector decarbonisation [84–86]. However, even after two decades of discussion, only one small pilot plant in Abu Dhabi is currently operating a blast furnace with CCUS, the captured gas is used for enhanced oil recovery, and the first analysis of the plant, published recently, states that even if the project were operating at full capacity (which is uncertain) it is far from being aligned with the targets of the Paris Agreement [87].

Hydrogen technologies are an emerging alternative for primary steel production, with recent evidence suggesting this can be economically competitive when combined with high-quality iron ore, low steelworker wages, and abundant cheap renewable electricity [53]. However, as discussed in Section"Zero-emissions energy", the availability of green hydrogen for zero-emissions steel production will be limited by infrastructure development and the availability of zero-emissions electricity [88]. Therefore, with neither blast furnace nor hydrogen routes likely to scale by 2050, primary production of steel is unlikely to be compatible with a realistic view of delivering zero-emissions in the UK by 2050.

However, a third of global steel production today is via the scrap-based electric arc furnace (EAF) route with 100% recycled feedstock [89]. Running on electrical power, which can be decarbonised, this production process is compatible with a realistic view of delivering zero-emissions in the UK by 2050. Considerable investment is required to convert UK steel manufacturing to EAFs, which currently only produce 22% of UK steel (compared with 40% in the EU) but during 2023, the UK government has demonstrated the political will to support this change. In September 2023, the UK government offered £500 m to support the conversion of BOF to EAF steel making at the Tata Steel facility in Port Talbot, Wales [90] and a similar scale of investment in Scunthorpe [91]. Around 10 million tonnes of scrap steel are collected annually in the UK, of which around 2 million tonnes are used in existing domestic EAFs and the remaining 8 million tonnes are exported [9]. Despite known benefits from recycling, including reducing waste and reducing prices [92], the remainder of UK scrap is currently exported, and a similar mass of steel is then re-imported as components or products. Although current recovery rates from demolition sites in the UK are 99% for structural steelwork and 96% for all steel construction products [93], the transition to an zero-emissions economy in the UK will increase domestic demand for scrap, incentivising new UK-based metal sorting and processing facilities, but without significant increases in recovery rates.

The *variety* of steel products that can be manufactured by recycling is limited by scrap quality [24]. Although saving 60–75% of energy use [94], recycling is often in reality “downcycling” as impurities, such as tin and copper, accumulate in each steel recycling cycle [95] causing hot shortness which constrains processing [65]. Compared to other applications (e.g. car panels), steel used in construction can tolerate a greater level of impurities, so often has a higher recycled content than average [24]. As a result, the feasible global supply of recycled steel within a Paris-compliant carbon budget will only meet 58–65% of the demand that would be expected in 2050 in the absence of climate change [16].

Direct reuse of steel components, without remelting, uses far less energy than recycling [[96] so as energy constraints cause price rises, construction steel will increasingly be supplemented by direct re-use of steel accumulated in products and infrastructure [97]. A growing body of literature investigates this opportunity for reuse and the significant environmental benefits that it might create [98]. However, little reuse occurs at present [99]; whilst 93% of all heavy structural sections and tubes are recycled in the UK, only 7% are reused [72]. The main barrier to greater steel reuse is the collection, storage, testing and certification of used steel components [100]. There is some evidence that standardisation, pre-demolition surveys and design for deconstruction could increase steel reuse in the UK [69]. But it is, as yet, unclear what quality and quantity of reused structural steel could be available in 2050.

In summary, the decarbonisation of primary steel production relies heavily on the uncertain deployment of CCUS and hydrogen, which are unlikely to be economical by 2050. However, the secondary production of steel via EAF steelmaking, powered by zero-emissions electricity, and using 100% recycled feedstock, is compatible with a realistic view of delivering zero-emissions by 2050. A transition to this mode of production will increase the demand for scrap, incentivising the exploitation of new sources and UK-based metal sorting and processing facilities, whilst still heavily restricting the total supply of steel.

### Aluminium

**Fig. 7:**
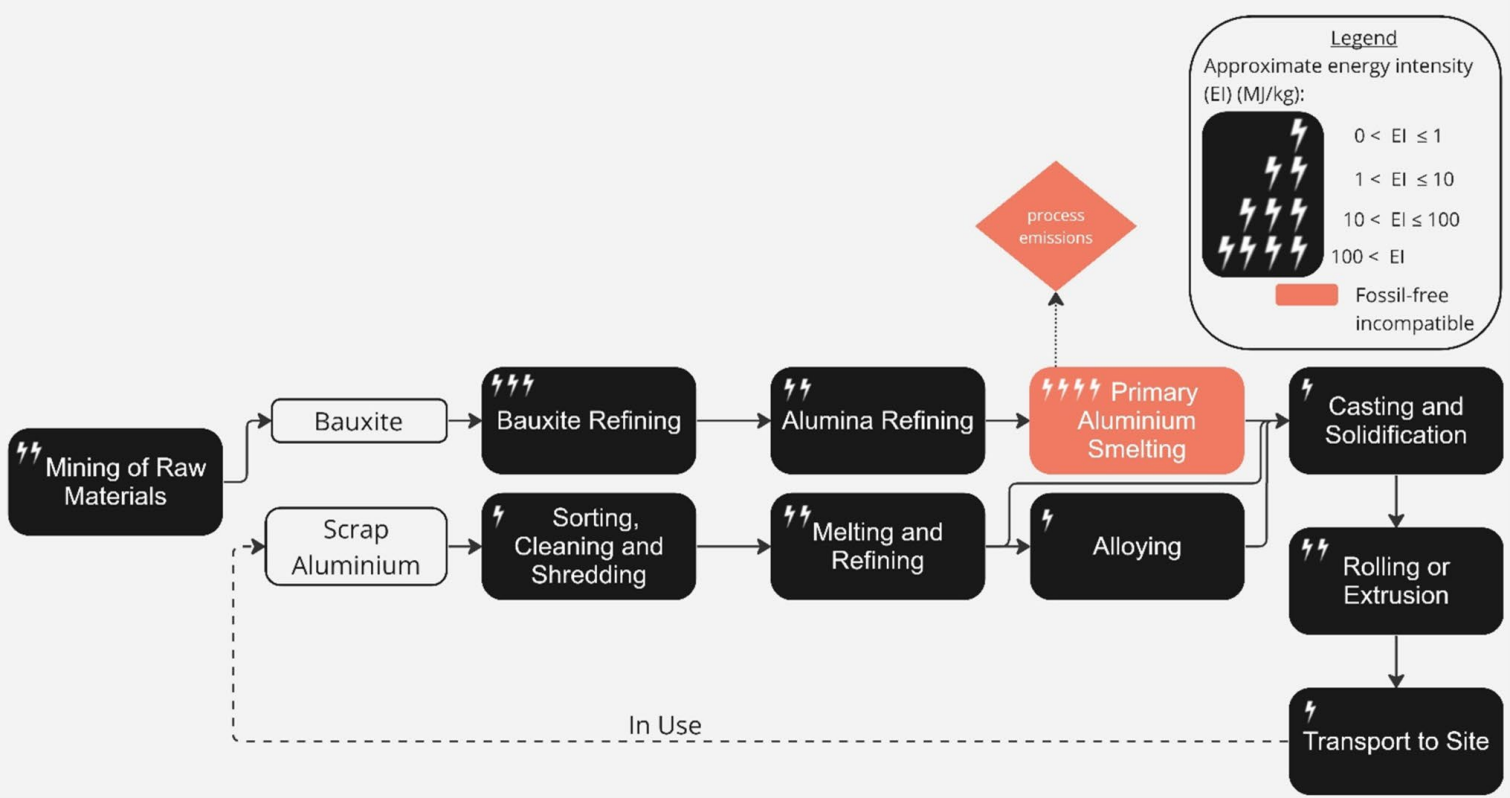
Process map of primary and secondary (via ‘Scrap Aluminium’ route) aluminium production, adapted from (Cushman-Roisin et al. 2021). The approximate energy intensity of each process in the production chain, and the process’ compatibility with a realistic view of delivering zero-emissions by 2050, is indicated (see figure legend) [19]

Aluminium is primarily used in construction for building cladding systems, in high-rise and lightweight structures, and can be used to create intricate architectural shapes with high material efficiency [101]. Aluminium is favoured for its corrosion resistance and low specific weight. There are four major stages to producing primary aluminium, as described in Fig. 7: mining of bauxite ore, with a high content of aluminium in the oxide alumina; bauxite refining to alumina in the Bayer process; alumina reduction to liquid aluminium via smelting in the Hall–Hérault electrolysis process; casting [102].

All four steps release emissions due to electricity use, and smelting is particularly energy intensive, leading to 61% of total emissions from primary aluminium production [105]. Globally, ~ 55% of the electricity needed for aluminium smelters comes from coal or natural gas [106]. Therefore, directly addressing the supply of electricity is key to decarbonisation. To mitigate the intermittent nature of wind and solar power for smelting, some producers resort to hydropower to generate significant and consistent loads [107]. However, this option faces potential growth limitations, regional specificity, and susceptibility to droughts. The introduction of small modular nuclear reactors (SMRs) could have a high potential impact yet currently has a low market availability to date due to regional regulatory stance on nuclear (e.g. disaster risk and nuclear waste) so it is unlikely that this will be available at scale by 2050 [107].

The process also releases some process emissions, in particular when CO_2_ is released at the anode of the electrolytic cell (around 11% of total emissions from primary aluminium production). These emissions could be reduced and potentially eliminated if carbon anodes were replaced with inert designs which release only oxygen in use [108]. The IEA’s Net Zero Scenario [109] sees inert anodes used for around 7% of primary production by 2030. However, they have been under development for several decades and are yet to reach commercial maturity. As of yet, an adequately effective anode material has not been identified, so is unlikely to be ready for large-scale deployment in the near future [110].

Recycling aluminium by re-melting is therefore the most feasible route to zero-emissions future production. Recycling perfectly sorted aluminium of a single alloy requires only melting and casting. Recycled, or secondary aluminium can offer the same quality as primary aluminium, and the industry claims potential energy savings of up to 90–97% compared to virgin production [12]. However, in practice, savings are somewhat lower as most recycling involves other processes such as de-lacquering and some virgin aluminium is mixed with re-melted scrap to control alloy composition.

The UK is a global leader in aluminium recycling, generating an estimated 1.4 m tonnes of aluminium scrap per year of which 0.5 m tonnes is exported [12]. With increasing demand for zero-emissions aluminium in construction [111], scrap supplies could be insufficient to meet demand which will increase prices, and as UK collection rates are already high, supply will likely be short of the demand that would occur without climate constraints [112]. In 2012, Allwood J. and Cooper D. found that aluminum building components may also be reused in the future: window frame and curtain wall extrusions could be more standardised and be installed with a connection that would allow deconstruction without compromising future seal quality [113]. However, to date, there are limited real-life examples of aluminium components being re-used in new build construction in the UK.

To sum up, the primary production of aluminium is incompatible with a realistic view of delivering zero-emissions by 2050 due to the unavoidable process emissions, making re-melting of recycled material the preferred zero-emission compatible pathway. As for steel, this will lead to increased demand for scrap and higher prices due to a restricted supply of the material.

### Structural glass

**Fig. 8:**
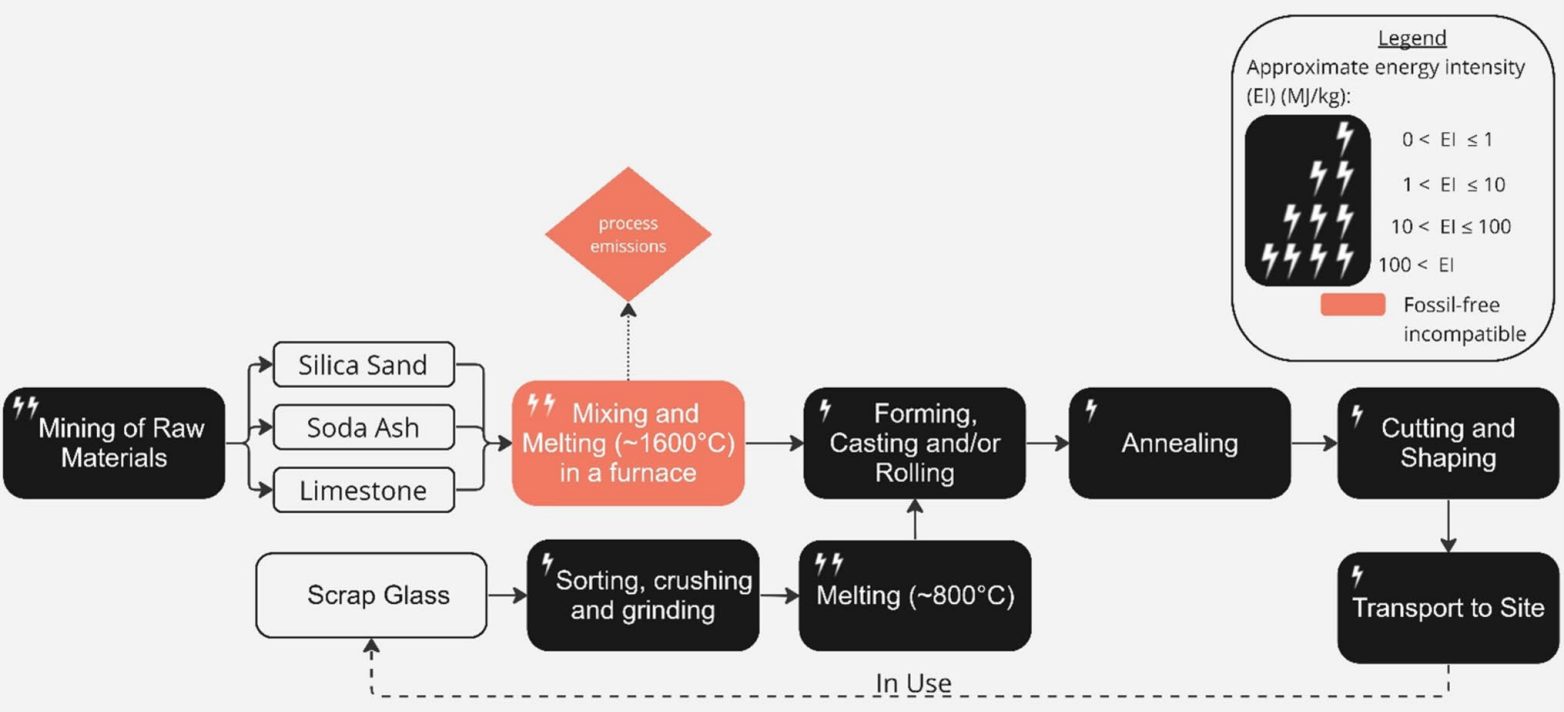
Process map of primary and secondary (via ‘Scrap Glass’ route) glass production, adapted from (Schmitz et al. 2011) and (Debrincat et al. 2018). The approximate energy intensity of each process in the production chain, and the process’ compatibility with a realistic view of delivering zero-emissions by 2050, is indicated (see figure legend) [103, 104]

Structural glass used for doors, windows or roofs, is produced by melting sand, limestone, and soda ash (Fig. 8). In the UK, three manufacturers produce around 750,000 tonnes of glass each year, mainly for use in buildings [114]. Primary glass making requires temperatures of ~ 1600 °C, typically achieved using natural gas. The heat could be supplied by electrification although this would greatly increase electricity demand while zero-emissions supplies are constrained [27]. However, primary glass production leads to process emissions which cannot be avoided through electrification. Replacing all virgin feedstock with recycled glass (cullet) would both avoid process emissions and, as cullet melts at a lower temperature than raw materials, for every one tonne of cullet used in the manufacture of float glass, 300kWh of energy is saved [103]. Currently, the recycled content of flat glass produced in the UK is between 20%–30% and the main barrier to increasing this is the availability of recovered glass of acceptable optical quality [27]. The engagement of all stakeholders involved in building renovations and demolition will be required to increase the quality and quantity of cullet supply [114].

Alternatively, re-using glass products, without melting, would eliminate almost all emissions. At present re-use of flat glass is rare, due to the technical difficulty and cost of removing window glass from buildings without damage, increasing quality requirements of new buildings and the challenge of matching demand to supply [103].

As a result, the need for complete circularity will somewhat constrain the supply of glass, and the energy-intensive process will encourage direct re-use and reconditioning of glass panels from demolition sites.

### Timber

The idea of substituting carbon intensive materials such as cement and metals with regenerative resources from the biosphere has attracted much attention as a means to decarbonising the built environment [115, 116]. Timber is the most prominent bio-based construction material, with a long history of structural use for piles, frames, cladding and roofs [17]. Engineered timber products, such as Cross-Laminated Timber (CLT) and Glulam, are strong, stiff and consistent, allowing rapid construction, and enabling full or hybrid timber structures [117].

Timber typically has low life cycle emissions compared to other structural materials. For example, in low-rise residential buildings, timber framed structures cause approximately 30% less embodied emissions than concrete block cavity wall construction [118] and a greater reduction of ~ 60% can is seen for larger structures that use engineered timber products such as (CLT) instead of concrete [119].

UK timber consumption is currently lower than in other countries such as USA, China, Japan and Germany [120]; structural timber is used for just 22% of new housing [121]. The UK Government promotes rapid expansion of the industry as part of its net zero strategy [122] in part supported by aims to increase woodland coverage from 13% of UK land at present to 17–19% by 2050. However, as the UK currently imports 80% of its timber (making it the third largest net importer of timber and wood products in the world) [123], the UK faces a critical challenge in increasing the short-term supply of sustainable timber products for construction.

**Fig. 9:**
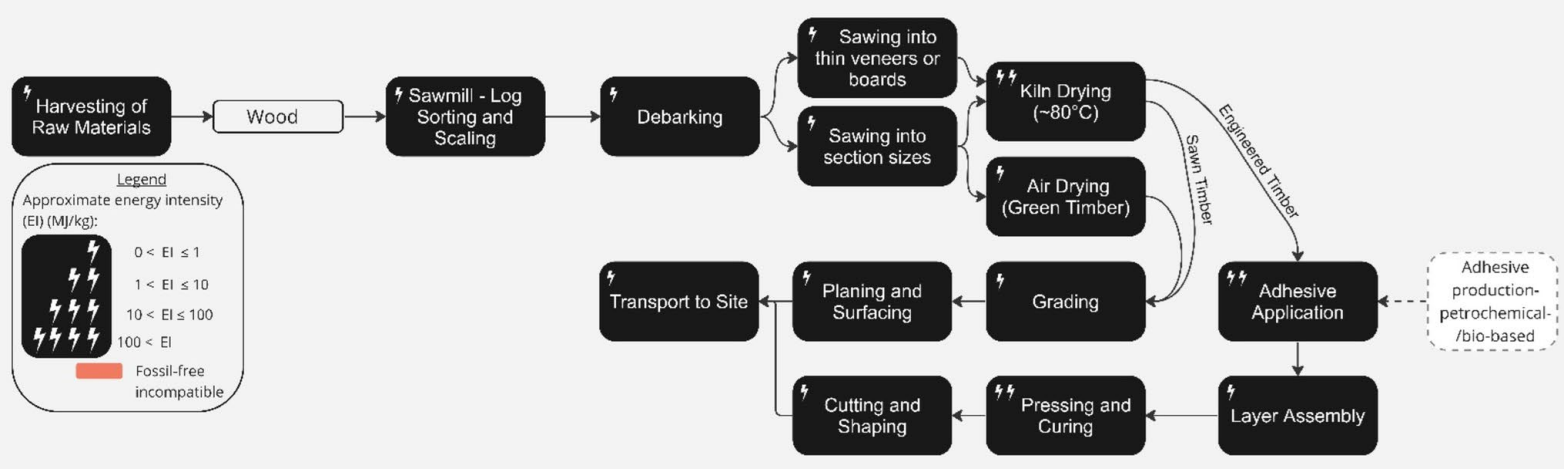
Process map of sawn timber and engineered timber production, adapted from (Mishra et al. 2022), (Hart et al. 2021), (Konopka et al. 2021) and (KLH Massivholz GmbH. 2019). The approximate energy intensity of each process in the production chain, and the process’ compatibility with a realistic view of delivering zero-emissions by 2050, is indicated (see figure legend) [125–127].

Achieving zero-emissions requires electrifying the entire timber production chain, as described in Fig. 9. The largest proportion of emissions comes from transport (55% from both territorial and overseas) due to the use of diesel to power forestry equipment and vehicles. In the immediate future, continued imports will require long-distance transportation from disparate European locations, perpetuating diesel emissions. However, early innovators such as Hanson have begun to use electric HGVs to transport OSB, MDF and plywood products across the UK [124].

Unlike materials with process emissions, non-transport emissions of timber production can be mitigated through the electrification of forestry, limbing, debarking, and sawing. Case studies of electrically powered forest harvesters and zero-emissions timber production in sawmills in Finland are provided in the Timber Development UK ‘Net Zero Roadmap’ [128], however, there is very limited evidence of adoption in the UK to date. Energy-intensive processes such as kiln-drying in production mills are typically powered by natural gas or biofuel but could be electrified relatively easily [23] since they require far lower temperatures (around 100 °C) than kilns for other materials. For engineered timber products, new bio-based adhesives must be developed to avoid process emissions from petrochemical-based glues [129] or new joining methods such as dowel laminated timber (DLT) could be expanded [130].

In 2020, the UK generated around 4.5 million tonnes of waste wood [131]. As with steel, timber can be reused [132]. However, glue use and destructive disassembly currently inhibit reuse which promotes downgrading used wood into woodchip or for fuel. The scale of future supply of waste wood for structural timber has as yet not been estimated.

Given these points, it is clear that the decarbonisation of timber production is relatively straightforward and therefore could feature as a key alternative to energy-intensive structural materials such as steel in the near future, albeit constrained by a limited supply of resources from sustainably managed forests.

### Earth

**Fig. 10:**
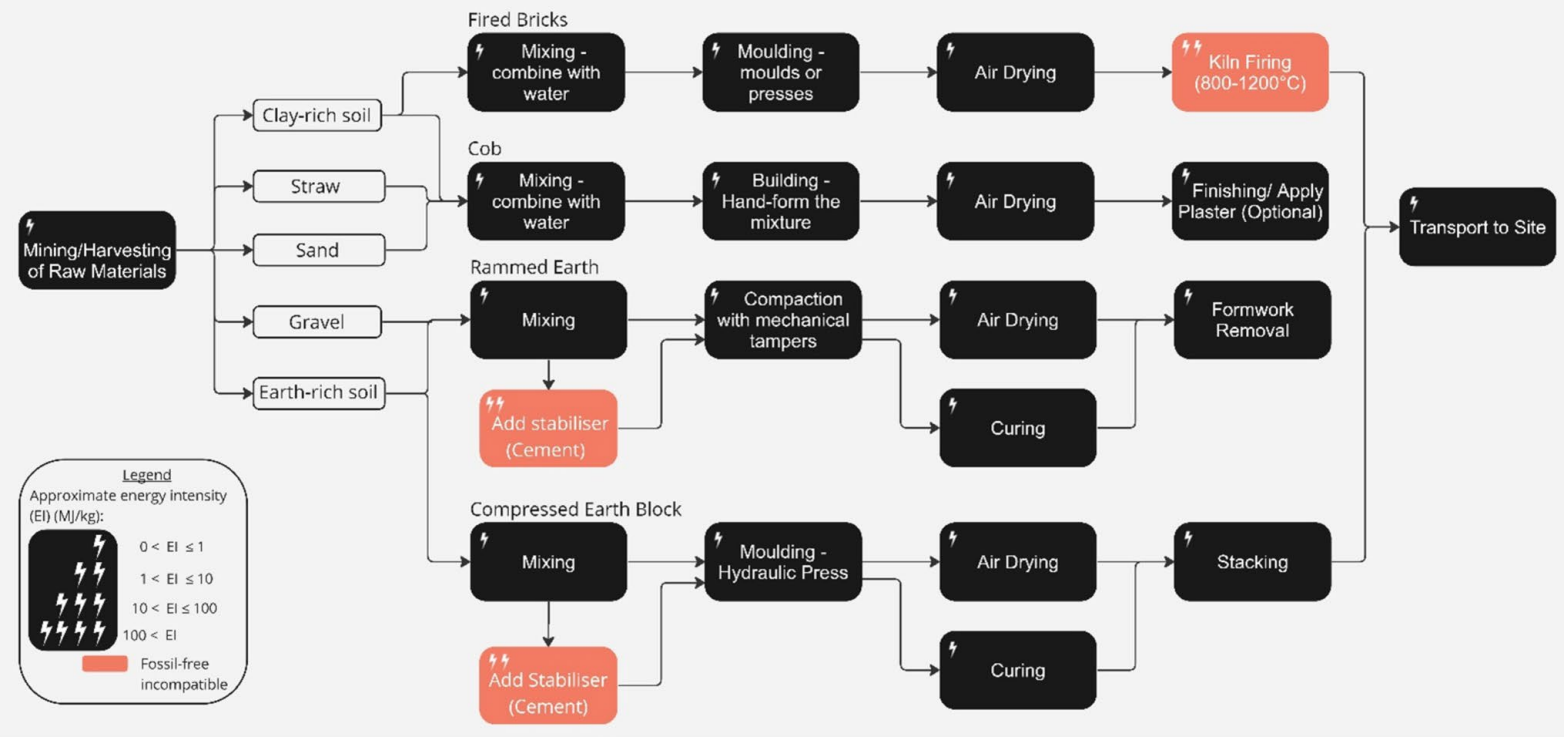
Process map of fired brick, cob, rammed earth, and compressed earth block production, adapted from (Fernandes et al. 2019), (Ben-Alon et al. 2019), (Kariyawasam et al. 2016), (Maskell et al. 2014) and (Morel et al. 2021). The approximate energy intensity of each process in the production chain, and the process’ compatibility with a realistic view of delivering zero-emissions by 2050, is indicated (see figure legend) [140–145].

Today, fired earthen materials, such as clay bricks, are typically used in a non-structural capacity, yet the UK still produces over 2 billion bricks annually [133], generating 1.8 m tonnes of carbon emissions and accounting for 6% of the total material mass in UK construction [28]. These emissions arise both from the use of fossil fuels to produce high firing temperatures (~ 1000 °C) [134] and from process emissions released during firing which can cause 30–60% of total emissions, depending on the composition of the minerals and the soil-source geology [135]. Firing can be electrified, albeit at a high energy cost, and, according to one study, existing technologies could lead to electrification of 78% of brick production in the EU [136]. Other than electrification, further reduction in energy use is unlikely without fundamental improvements in materials and processes [137] or CCUS to reduce emissions from fuel combustion [138]. Therefore, the future supply of zero-emissions fired bricks is likely to be limited by the availability of zero-emissions electricity [139] (Fig. 10).

**Fig. 11:**
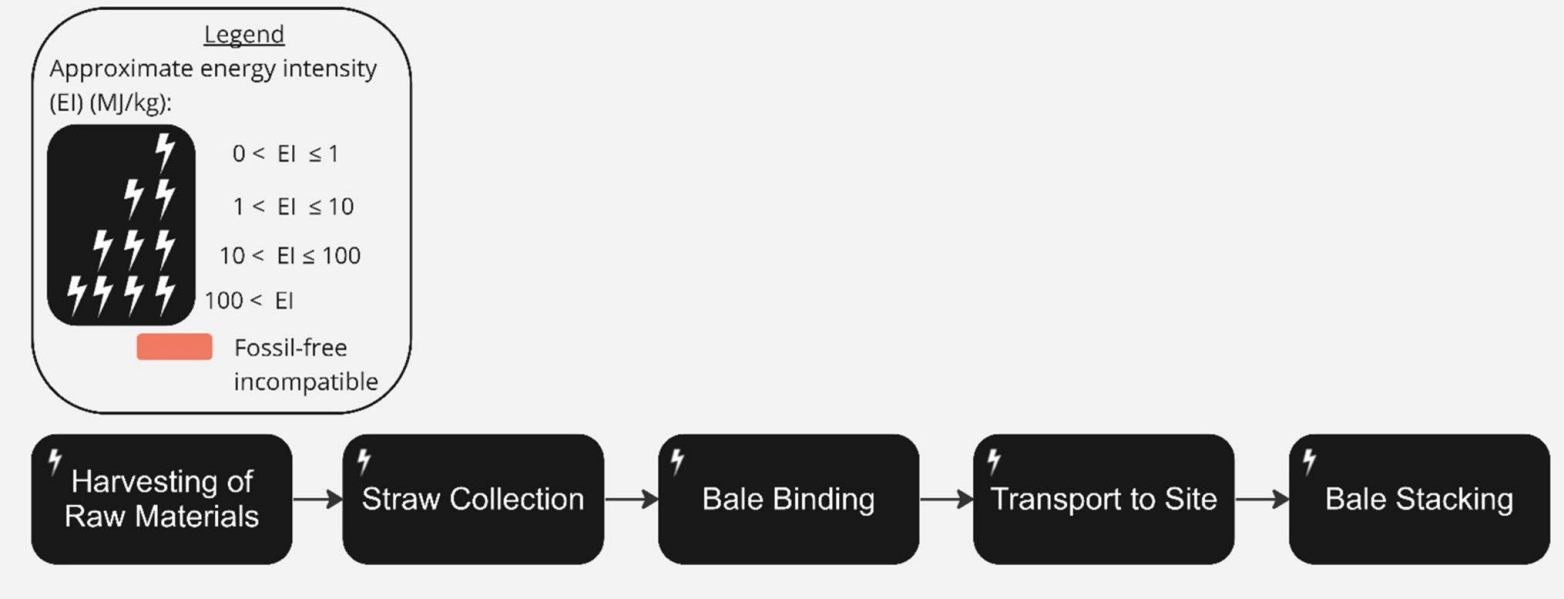
Process map of strawbale production, adapted from (School of Natural Building. 2021). The approximate energy intensity of each process in the production chain, and the process’ compatibility with a realistic view of delivering zero-emissions by 2050, is indicated (see figure legend) [139].

Whilst earthen building materials today are primarily fired, earth is integral to many earlier construction technologies which do not require heat or chemical change but are instead manipulated through variations in moisture content [146]. Raw earth construction is rare in the UK today and typically limited to one or two-story load-bearing wall construction. Use is constrained by a lack of clarity about construction methods [17], high labour requirements [17], durability concerns of earthen material in wetter climates [147], variable compressive strength [148] and failure to achieve strict thermal requirements [149]. However, research is now updating techniques to satisfy UK building regulations and create industrialised and mechanised forms of earth building. Some of the more promising techniques [150] include rammed earth, cob, CobBauge [151] and Compressed Earth Blocks (CEBs), as well as Light Earth Construction (a hybrid design combining a load-bearing structure, typically timber, filled with earth slip and straw) [146, 152]. The production processes of these earthen building techniques are described in Fig. 11 (the production of CobBauge and Cob are assumed to be the same up until construction stage [151]).

Those earthen materials which often have stabilisers added to them to improve durability, such as rammed earth [153] or CEBs, are not compatible with a realistic view of delivering zero-emissions in the UK by 2050 since there are currently no zero-emissions options available at scale for replacing the cement or lime binders. However, unstabilised earthen materials are compatible as the processes and tools used in production, need little if any heating and do not release process emissions [154]. Expanded zero-emissions use depends on the electrification of mechanical equipment such as excavators and crushers which mix soil to a homogeneous consistency [155] and transport [156]. It should be noted that an increase in earth building would require a cultural shift to accept higher labour costs and more frequent maintenance.

Ultimately, the primary production of earth products is compatible with a realistic view of delivering zero-emissions by 2050, so long as the production does not involve kiln-firing or the addition of cement-based stabilisers. In this context, earth also performs well as a circular material as it can be returned to the soil at end of life [145]. Further research and development are crucial to unlocking the full potential of these materials and integrating them into modern construction practices.

### Straw

Straw is an agricultural fibre made from crops such as wheat and oats and has been used extensively in construction around the world since prehistoric times [157]. Wheat is grown all over the UK but predominantly in central and eastern locations [158]. Straw can be used in thatch and has good insulative properties which enable its uses as infill insulation [139], load-bearing straw bale construction [158] and more recently, prefabricated external wall systems [159]. Development of ‘industrialised’ straw bale construction has led to quality assurance [118] and companies such as Modcell and EcoCocon now use straw bale technology to make standardised panels, demanding very little primary energy [160].

The production process, described in Fig. 11, involves straw growth (tillage, fertilisation, and sowing) which in LCA analyses is typically considered a by-product of grain production [161], followed by collection (mowing and haying), baling and transport. Progress has been made in decarbonising all these processes [162]. GHG emissions from fertilisers (ammonium nitrate) could be reduced by one-fifth with currently available technologies [163]. Farming machinery can also be electrified [164], and as a result, for example, zero-emissions electrification of on-field tractor operations could reduce annual CO_2_ emissions by 69% in 2030 and 97% in 2050 [165]. At end of life, straw can be returned to the soil and composted [166].

Strawbale construction is compatible with a realistic view of delivering zero-emissions in the UK by 2050, but the UK lacks a formal supply chain and there is a shortage of data on the quantities of straw produced in the UK, and the split of use between current applications (e.g. use in animal bedding/feed, in-field protection of high-value crops, co-fired energy production, incorporation into the soil).

### Stone

Much of the world’s architectural heritage is testament to the successful use of stone in construction over millennia. The use of stone buildings in Scotland date back to 4000BC when farming created a need for more permanent structures [169]*.* The construction of these buildings was labour-intensive, with sandstone typically sourced from local quarries to avoid transport. In recent decades there has been a resurgence of interest in natural stone as a low-carbon material for modern architecture [170]. Today, technologies such as form-finding [171] and post-tensioning [172] can produce highly optimised structures.

**Fig. 12:**
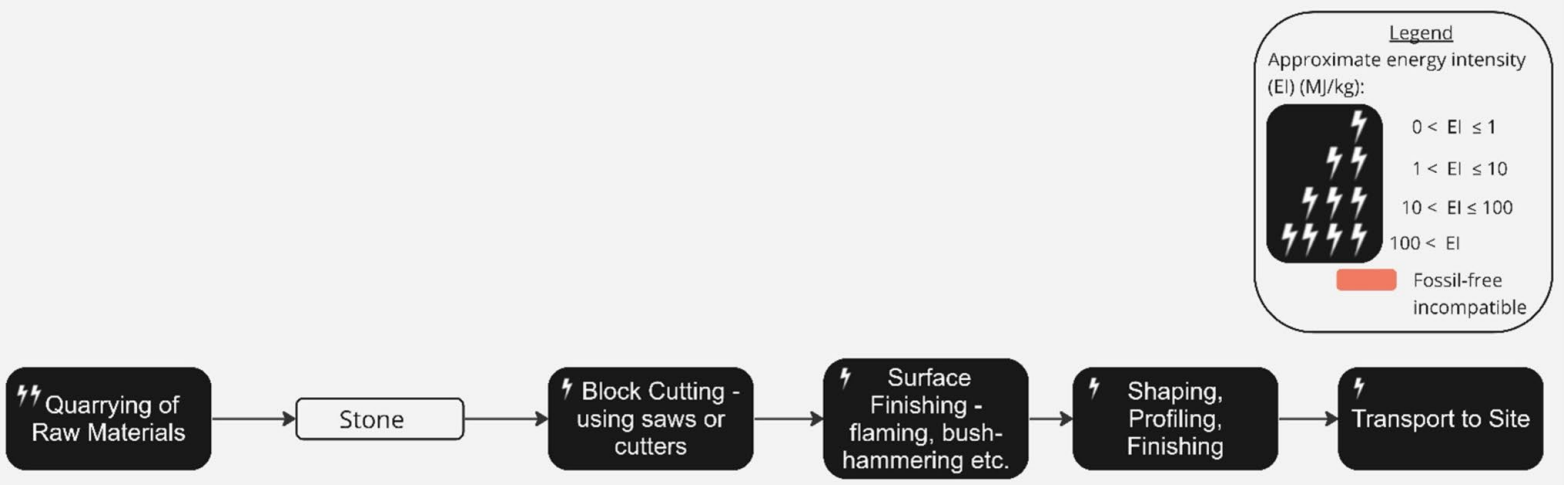
Process map of dimensional stone production, adapted from (Crishna et al. 2011). The approximate energy intensity of each process in the production chain, and the process’ compatibility with a realistic view of delivering zero-emissions by 2050, is indicated (see figure legend) [167, 168]

To produce dimensional stone for modern construction, as described in Fig. 12, diesel and petrol are required in quarries for drills and excavators [173]. Decarbonisation of explosives is key to structural stone production becoming zero-emissions compatible, specifically the production of ammonium nitrate which is essential for dynamite [174], but which could be avoided for example by re-adopting past practices in which stone was drilled, wedged and split into blocks by hand [175]. In stone yards, energy is needed for stone-processing machinery, pumping water, extracting dust and transporting the finished stone, but all of these can be feasibly electrified [167].

Previous research indicates that, due to the energy-intensive mobilisation requirements of heavy stones, the transportation distance can greatly impact the total embodied emissions of structural stone production. For example, assuming average French limestone densities and typically implemented thicknesses of 24 cm, the total embodied impact of a masonry wall (1m^2^) is shown to double for truck-transit distances increasing from 60 to 579 km, such that the transportation to the construction site would go from representing 12% to 56% of the total impact [168]. Although, as discussed in Section "Zero-emissions transport", road freight can be feasibly electrified in the near future, the supply of stone is likely to be constrained by what can be sourced locally due to high energy costs for long-distance haulage.

Load-bearing stone has strong potential for reuse at end of life, as seen in Ancient Roman, Greek, and Egyptian practices of repurposing masonry—known as *spolia* (Latin for"spoils")—from demolished buildings. Cleaning and reusing existing stones or bricks required far less labour than quarrying or producing new ones [177].

Stone construction is compatible with a realistic view of delivering zero-emissions by 2050, and although its use has a long legacy in the UK, the scale-up potential of structural stone use in the UK today, considering suitable stone reserves and present-day socio-economic and ecological barriers, has not yet been studied.

### Lime

Lime has been used in the UK for bedding and pointing mortars, renders and internal plasters, and in the construction of floor slabs since the Romans introduced the technology 2,000 years ago [178]. Until the 1920 s, lime-burning, although small scale, was widespread. Over the last few decades, lime has been rediscovered for use as a binder in bio-based products such as Hempcrete, a hemp-lime mix used in conjunction with a structural (timber) frame as a load-bearing system.

**Fig. 13:**
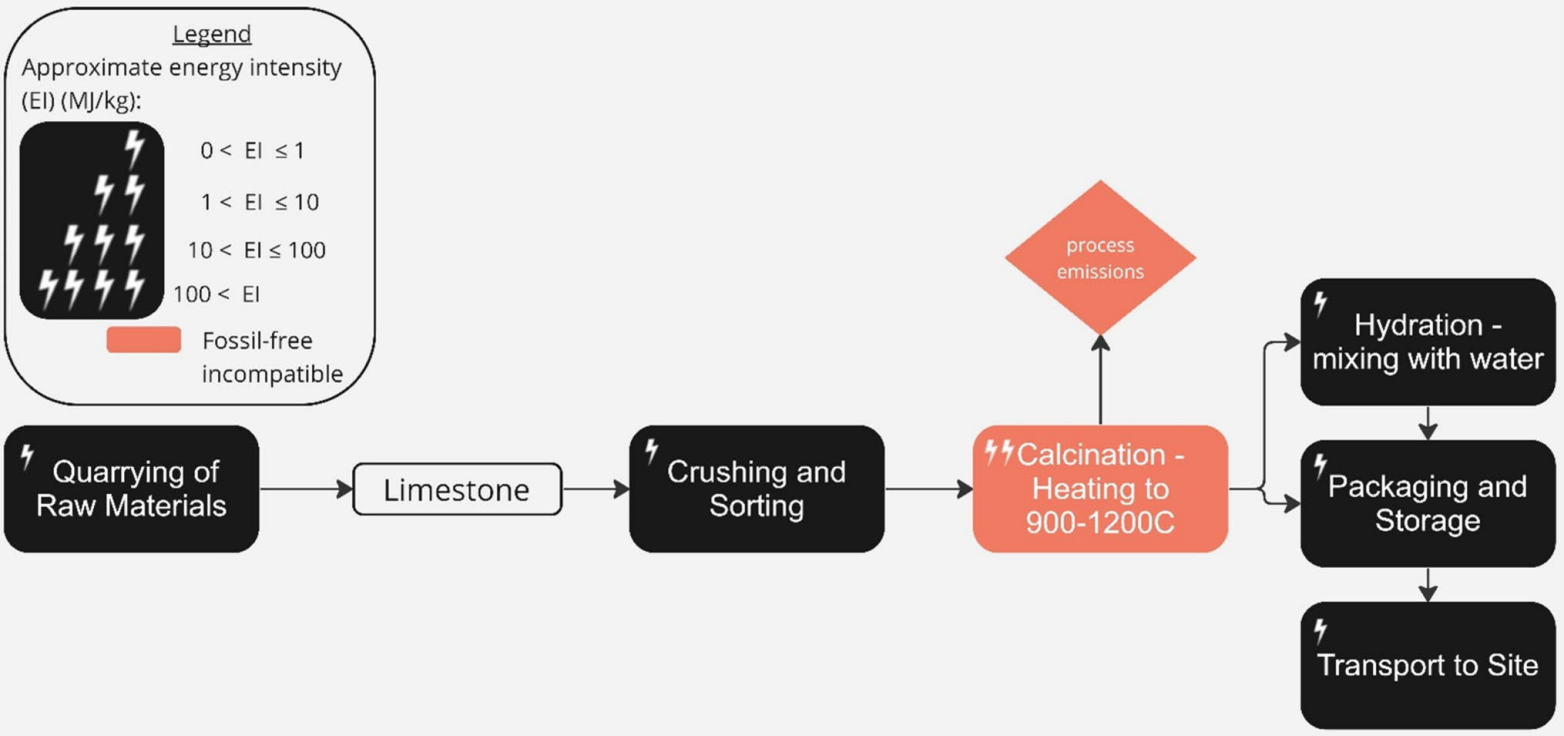
Process map of lime production, adapted from (Simoni et al. 2022). The approximate energy intensity of each process in the production chain, and the process’ compatibility with a realistic view of delivering zero-emissions by 2050, is indicated (see figure legend) [176]

The production of lime, as described in Fig. 13, requires calcining limestone (mainly CaCO_3_) at ~ 950 °C to produce lime (CaO) using fossil fuels. This process is energy intensive and leads to CO_2_ emissions from mineralogical transformation (68%), fuel combustion (30%) and electricity consumption (2%) [179]. In addition to improving energy efficiency using vertical kilns rather than horizontal kilns, current roadmaps for emissions mitigation include fuel switching, kiln switching or possible CCUS [179]. However, as only one-third of the emissions are generated by fossil fuel combustion, and since about 95% of the total energy input is employed for calcination, this would lead only to small savings.

Lime hardens through carbonation, so some of the process emissions from production are reabsorbed relatively rapidly. Because of this, a recent report by the Mineral Products Association [11] claims that the UK lime sector can absorb more CO_2_ from the atmosphere than is emitted in production and help to offset other hard-to-abate emissions, although this relies on both the use of biomass fuels and CCUS to mitigate production emissions. However, a recent study commissioned by the European Lime Association (EuLA) shows that, across major applications of lime, only around 33% of the process CO2 emitted during lime production is captured permanently during use [181].

Therefore, due to a reliance on CCS to capture residual emissions, the production of lime for use in construction does not fit well within the zero-emissions resource pool.

## Discussion

This review of key construction materials identified two important issues that have had insufficient attention in the literature.

### Zero-emissions primary production of key construction materials will be constrained until 2050

Primary production of many key materials including concrete, steel, aluminium, structural glass, fired and/or stabilised earth bricks, and lime, is incompatible with a realistic view of delivering zero-emissions in the UK by 2050. Continued use of these materials will therefore cause emissions that could only be mitigated through genuine offsets, such as landscape restoration. However, the world faces a “global land squeeze” [182] from pressure to increase food and wood production [183], while expanding urban areas threaten natural habitats. Land-use change has probably contributed up to a third of anthropogenic carbon in the atmosphere to date [180] and further land-use changes will likely expand this contribution, for example through deforestation [184] or melting permafrost [185]. Therefore, offsetting residual emissions via landscape restoration is a high-risk strategy.

In contrast, recycling and re-use of these materials can be compatible with a realistic view of delivering zero-emissions in the UK by 2050. Nevertheless, while recycling can be electrified, the processes are energy intensive, so will be constrained by the availability of zero-emissions energy which may meet only 60% of anticipated demand [44]. Furthermore, as long as the demand for construction materials grows, the supply of scrap will only meet part of the demand (due to the long lifespan of buildings and the practical limits of recycling) [186].

Direct material reuse is the least energy-intensive mode of material production and could become increasingly attractive in an energy-constrained near future. However, re-use is not currently standard practice and new supply chains must be developed to match supply to demand [132]. At present, less than 1% of UK construction and demolition waste is reused [187], and it is unclear how much material will be available for reuse in future. Some stock analyses have been published, for example, to estimate timber stocks in a London Borough at around one tonne per dwelling [188] and attention is now given to dynamic material stock models [189] and the use of GIS tools for spatial representation of stocks [190]. However, more work is required to anticipate future quality and quantities of material available for recycling and re-use, and there remains a challenge in understanding how to balance the many competing claims on the energy and land resource pools in a 2050 zero-emissions economy.

### Overlooked potential: primary material production compatible with the zero-emissions resource pool

Some more traditional materials such as timber, unfired and unstabilised earth, straw, and stone are compatible with a realistic view of delivering zero-emissions in the UK by 2050 but have largely been overlooked to date. Typically, these materials require little processing or high-temperature (< 100 °C) heating as they are taken directly from nature [191].

Like many major economies, demand for housing in the UK outstrips supply [192] an estimated 300,000 new homes per year are required [193]. Recent research by Drewniok et al. [194] predicts that, based on anticipated population trends, the domestic building stock will grow 11% to reach almost 2.9 billion m^2^ by 2050 [187]. Therefore, there is scope for increased use of these natural materials. However, it is unclear how much of each material could be required, and making such an estimate would require a material database for different UK building typologies [28].

New developments in processing could increase the quality and quantity of supply. For example, quarries could produce hundreds of stone beams and columns per hour and automatic X-ray fault identification could improve the reliability of stone products, reducing safety factors and permitting slender, high-strength elements [195, 196]. These standardised products could be used locally, and be re-used indefinitely, with the development of appropriate construction approaches.

Concurrently, it is difficult to expand the primary supply of natural or biomass-derived materials. Their sourcing can require more land than existing common materials, so competes with other uses, established by complex patterns of policy, tradition, historical ownership and cultural association [197]. Competition comes from farming food and feedstock [198], housing [199, 200], infrastructure including roads, water and energy supply [198, 201], mineral extraction [202, 203] and carbon sequestration [204]. Yields can be increased with more labour or fertilizers [205]*,* but expanded use of biomass for construction can only reduce GHG emissions if sustainable forest and agricultural management are practised [206]. Land-use change is also considered to be the primary contributor to biodiversity loss in land-based environments [207]. A systems approach is required to examine how the environmental impact of land use by the construction sector can be balanced against other goals. Several studies have begun to answer the question of construction and land use [121, 201–203], yet the land-use changes arising from a shift to zero-emissions resource construction have not yet been fully articulated [199]. Similarly, the social impacts of such a change remain under-explored [208–210].

Anticipating the scale of future zero-emissions material supply from virgin, recycled and re-used materials, is crucial to planning future construction strategy. Various methods for estimating and mapping the supply of resources can be found at various scales within the fields of food [211], agriculture [212], engineering [213, 214], land management [215, 216] and soil science [217, 218]. The majority of these methods involve bottom-up analysis using GIS to produce thematic maps based on total or regional resource availability and then quantifying an upper limit of resource availability**.** However, while some resource data, such as for soil or geology, is available in a spatially linked form [219], other resource data exists in purely numerical format [16].

Food systems research [220, 221] creates a precedent for determining the size of a land-dependent resource pool. For example, the resource inputs required for a global transformation to sustainable food production for 10 billion people by 2050 have been estimated [239] demonstrating requirements to reduce yield gaps and adopt land management practices that shift agriculture from a carbon source to sink. A similar approach has quantified the required material, embodied land and carbon storage for construction with four key bio-based materials (hemp, straw, timber, bamboo), construction activities until 2050 [213]. While these attempts provide a basis for evaluating policies regarding both land-based resource strategies and trade systems, integration of such an approach with comprehensive projections of a full array of construction materials (including those from demolition) could usefully support better planning for construction with a zero-emissions resource pool. For example, detailed modelling of timber, stone and re-use supply chains may support a better understanding of the interaction between regulation, land use and other impact categories, while improving predictions of future end-of-life material supply.

## Conclusion

The key message of this study is that realistic deployment rates and holistic resource demands for novel primary material production technologies must be considered when formulating decarbonisation strategies for construction materials. The study has reviewed available decarbonisation strategies for zero-emissions material production in the UK by 2050 and explored which materials and their processes are compatible with a realistic view of delivering zero-emissions in the UK by 2050, or ‘zero-emissions resource pool’, as summarised in Table 1.

**Table 1:**
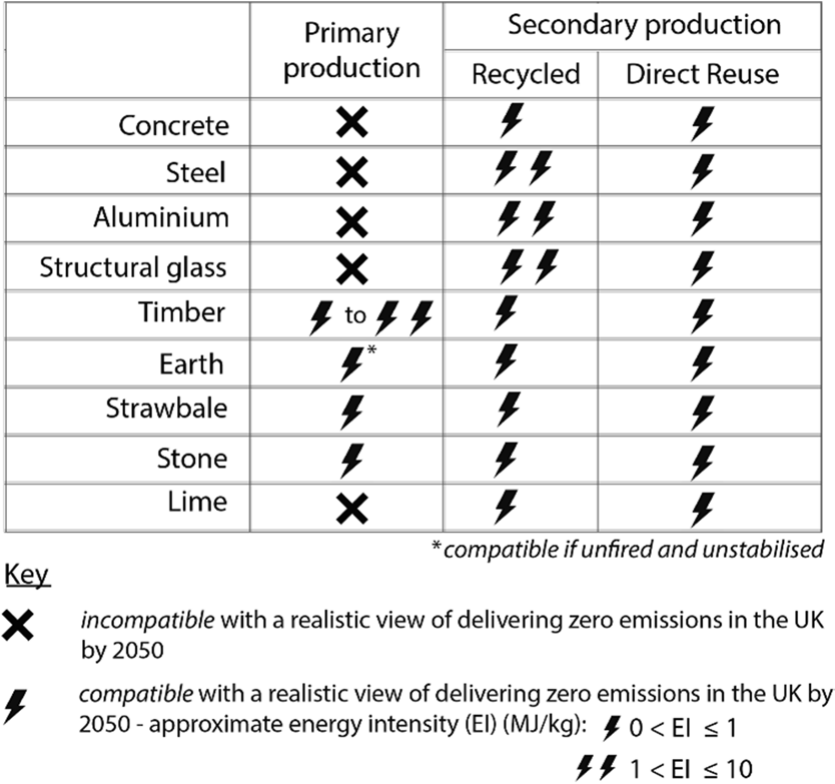
Summary table of the materials reviewed and their compatibility with the zero-emissions resource pool

The lightning icon indicates energy intensity and not recycling or reuse potential. For example, whilst the energy intensity for the direct reuse of materials is the same, the material quality, and practicalities and of reusing each material, varies greatly

Current industrial decarbonisation strategies rely heavily on technological infrastructure breakthroughs such as CCUS and green hydrogen, which have not yet been shown to work economically or at scale in the UK. This paper demonstrates that such strategies create substantial risks for the construction sector and argues that the low-risk route to zero-emissions construction in an zero-emissions future depends on transitioning to a building material palette sourced from a realistic estimate of the zero-emissions resource pool, which is likely to be constrained by zero-emissions energy capacity, limited end-of-life material supplies for recycling or re-use and by land-use constraints on natural material production. These findings emphasise the need to quantify the availability of these materials and establish the policies, innovation and entrepreneurship to deliver the services of construction from a much-restricted supply of construction materials.

## Data Availability

No datasets were generated or analysed during the current study.
